# *Carissa carandas* L. and Its Methanol Extracts Promote Hematopoiesis and Thrombocytopoiesis: A Sub‐Acute Toxicological Study in Experimental Rats

**DOI:** 10.1002/fsn3.71647

**Published:** 2026-03-13

**Authors:** Wisha Saeed, Muhammad Qamar, Raheel Suleman, Rai Muhammad Amir, Muhammad Waseem, Sheraz Ahmed Bhatti, Asher Abdur Rehman, Iqra Akram, Muhammad Zulqarnain Khan, Robert Mugabi, Tariq Ismail

**Affiliations:** ^1^ Department of Food Science & Technology, Faculty of Food Science & Nutrition Bahauddin Zakariya University (60800) Multan Pakistan; ^2^ Institute of Food Science & Nutrition Arid Agriculture University Rawalpindi Pakistan; ^3^ Department of Food Science and Technology, Faculty of Agriculture and Environment Islamia University of Bahawalpur Bahawalpur Pakistan; ^4^ Department of Pathobiology, Faculty of Veterinary Sciences Bahauddin Zakariya University Multan Pakistan; ^5^ Higher School of Medicine, Faculty of Medicine and Health Care Al‐Farabi Kazakh National University Almaty Republic of Kazakhstan; ^6^ Department of Food Technology and Nutrition Makerere University Kampala Uganda

**Keywords:** *Carissa carandas*, hepatocellular toxicity, iron deficiency anemia, nephrotoxicity, thrombocytopenia

## Abstract

*Carissa carandas—*a member of the family Apocynaceae, has gained tremendous attention from the scientific community for its impressive nutritional and health‐promoting features. There is little information available on the toxicological risks associated with consuming the fruit; therefore, the present study aimed to investigate the toxicity of the commercially viable doses of the fruit and its extracts in an experimental rat model. A 28‐day sub‐acute feeding trial was performed on male albino rats using 5%–10% supplementation of *C. carandas* dehydrated powder and methanol fruit extracts. Our results demonstrated a significant (*p* ≤ 0.05) increase in hemoglobin and hematocrit concentration, from 11 to 14 g/dL and 35%–46%, respectively. Hematological results further confirmed improved hematopoiesis and thrombocytopoiesis due to dispensing *C. carandas* and its methanol extract, anticipating a significant increase in platelet counts from 627 to 1090 × 10^3^/μL. The study's hepatic and renal histopathological findings also demonstrated no visible degenerative or inflammatory risks on sub‐acute administered doses of fruit powder and extracts. The results indicate that lower doses of *C. carandas* powder and extracts, that is, < 10% dietary supplementation, do not elicit any detrimental or toxicological effect in the rats, suggesting *C carandas* to be a valuable and promising candidate for developing value‐added functional foods to alleviate the risks of nutritional and other adverse health conditions.

## Graphical abstract

*Carissa carandas* powder and extracts improve hematological indices, for example, hemoglobin and hematocrits. Supplementation of *C. carandas* significantly enhances thrombocytopoiesis and platelets. Incorporation of *Carissa carandas* exhibited no toxic effects on liver and kidney histology.

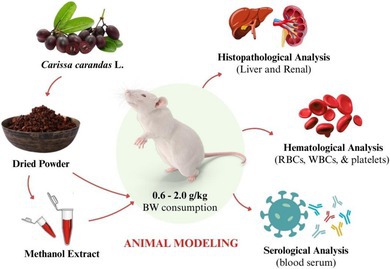

## 1 Introduction

Originating from the Himalayas, *Carissa carandas* L, a small berry‐sized fruit, is widely adopted and grown in different climatic conditions of India, Nepal, Pakistan, Afghanistan, Sri Lanka, Australia, Java, South Africa, Myanmar, and Malaysia. Because of its reasonable health promotion properties, *C. carandas* fruit, as a potential carrier of micronutrients, phenolics, flavonoids, peptides, and sugars, is widely employed in various cultures and traditions to develop products like curries, condiments, beverages, and sweetened recipes (Arif et al. 2020). Traditional medicine utilizes ethanolic extracts of *C. carandas* as a source of natural antioxidants bearing plausible hepatoprotective properties (Singh and Agarwal 2022). Similarly, the acetone extracts of mature fruits have been cited for exhibiting considerable anti‐inflammatory activity; likewise, freeze‐dried fruit juice and the residue powder have also been found to be promising functional food formulations due to their antioxidant, antiproliferative, and antidiabetic properties (Owatworakitb and Siriwongd 2023). In vitro and in vivo models showed that the fruit extracts exhibit anti‐inflammatory and anti‐insulin resistance activities that preserve pancreatic function and anticipate better glycemic control in diabetic models (Lailerd et al. 2023). Other documented uses of *C. carandas* fruit and its extracts include their cosmeceutical properties linked to anti‐inflammatory, anti‐wrinkle, and skin‐whitening features (Neimkhum et al. 2021).

*C. carandas* , known as Karonda in some cultures, have diverse food applications due to unique sensory attributes, nutritional composition, and bioactive compounds associated with functional properties. The fruit of *C. carandas* comprises 75% edible pulp; it is widely used in manufacturing value‐added food products of commercial significance, such as pickles, curries, and sweetened preserves of high culinary versatility and nutritional significance (Arif et al. 2016). Anthocyanin‐rich pulp fractions of *C. carandas* have been used to develop food colorants showing significant antioxidant properties. In addition, fruit extracts of *C. carandas* have been reported to increase shelf stability and consumer acceptability of fermented pork sausages, also known as Nham (Sueprasarn et al. 2017). In this regard, the antimicrobial properties of *C. carandas* extracts have been exploited in meat products against various foodborne pathogens and spoilage microflora, suggesting *C. carandas* as a carrier of natural preservatives for meat and meat products (Pilasombut et al. 2019). *C. carandas* extracts have been integrated into chitosan‐poly (vinyl alcohol) based biodegradable films to monitor freshness in beverages by leveraging the colorimetric response of *C. carandas* extracts to change pH and antioxidant activity (Singh et al. 2021). The efficacy of *C. carandas* extracts enriched chitosan coatings has been cited as productive in enhancing the shelf stability of tomatoes by reducing weight loss and maintaining other sensory characteristics of the fruit during storage (Aichayawanich et al. 2021). Furthermore, the fully ripened fruit of *C. carandas* is rich in bioactive natural pigments that may be used as a nutraceutical supplement and an ingredient of choice for developing naturally colored functional beverages (Krishna et al. 2007). As detailed above, varied applications of *C. carandas* fruit and its extracts in the food industry underscore its commercial significance and warrant studies on consumer safety for the fruit and its value‐added products.

*C. carandas* is a neglected and underutilized fruit that holds the potential to enhance food and health security by being rich in its nutrient pool and as a plausible source of health‐promoting bioactive compounds. With the increased demand for safer and healthier foods, incorporating neglected and health‐promoting fruit resources in food chains may help impoverished populations' nutritional and health security. Despite the benefits *C. carandas* holds for its consumers, consuming neglected fruits like this may not be without risks. Neglected berries and other fruits may carry antinutrients such as tannins and saponins that can impede nutrient absorption and bioavailability (Samtiya et al. 2020).

However, information on sub‐acute safety of *C. carandas* when incorporated into the diet at functional food levels is limited, because pharmacological potential of *C. carandas* was mostly reported from juices or solvent extracts, not whole‐fruit powder mixed in the diet (Anupama et al. 2014; Lailerd et al. 2023; Neimkhum et al. 2021; Owatworakitb and Siriwongd 2023). Also, although several toxicity studies of *C. carandas* leaf extracts (Shamim 2014; Rajesham et al. 2021; Madathala et al. 2022) have been published, they did not mimic repeated dietary exposure to the fruit powder at 5%–10% (w/w), and furthermore, while the wider toxicological literature on other botanicals has developed protocols to assess hematological outcomes (Abid and Mahmood 2019; Hor et al. 2012; Konan et al. 2007; Loha et al. 2019), data on *C. carandas* is scarce, especially concerning platelet indices and thrombocytopoiesis in a diet‐based context. The study was therefore conducted to investigate the potential toxicological effects of *C. carandas* fruit and its extracts, to further clarify the suitability of this underutilized fruit as a food or nutraceutical ingredient, and the study also extensively explores the effects of sub‐acute feeding of *C. carandas* fruit pulp and supplementation with its extracts on the physical and biochemical health indices of male albino rats. The data also provide the histopathological effects of *C. carandas* fruit and its extract, thereby excluding hepatic and renal toxicity as suggested in earlier studies (Arif et al. 2020; Lailerd et al. 2023; Neimkhum et al. 2021; Owatworakitb and Siriwongd 2023; Singh and Agarwal 2022), and generally, the study provides new information on the sub‐acute safety profile of *C. carandas* fruit powder and its extract, particularly with respect to hematological parameters and thrombocytopoietic responses in rats.

## 2 Materials and Methods

### 2.1 Sample Preparation

*C. carandas* fully ripened fruits were collected from the fruit garden of the Department of Horticultural Sciences, Bahauddin Zakariya University, Multan, during August–September 2023. Fruits were washed with potable water and manually deseeded. Seedless fruits were evenly spread over nylon woven trays and placed in a cabinet dryer (Pamico Tech, Pak) for drying at 40^ᴼ^C to the mean moisture content below 10%. Dried fruits were ground in a heavy‐duty grinder to mesh size below 100 μm and stored in an airtight container at refrigeration temperature for further use. Fruit extracts were prepared by mixing fruit powder with methanol at a 1:10 ratio. Extraction was performed by homogenous mixing of the powder in methanol on an orbital shaker for 48 h. Extracts thus prepared were filtered through Whatman filter no. 41, and the filtrates were subsequently passed through the Whatman filter no. 1 and later concentrated on a rotary evaporator. Residual levels of methanol in concentrated extracts were removed by evaporation in a hot air oven at 40^ᴼ^C.

### 2.2 Experimental Animals

The study and working protocols were approved by the Research Ethics Committee of the Faculty of Food Science & Nutrition, Bahauddin Zakariya University, Multan. Twenty male albino Wistar rats, 10–11 weeks old and weighing 152–185 g, were procured from the research animals rearing facility of the University of Lahore, Lahore‐Pakistan. Rats were acclimatized to the animal laboratory conditions for 7 days and fed on a control diet (Table 3). Animal room conditions were maintained with temperature between 25°C and 28°C, relative humidity ~70%, and a 12 h light and dark period. Animals were given free access to food and water during the entire feeding duration. Control and experimental diets were prepared following the rats' diet preparation guidelines and offered to the rats as pallets. After 1 week of acclimatization, a twenty‐eight‐day experimental feeding trial was conducted.

### 2.3 Sub‐Acute Toxicity Study and Sampling

OECD guidelines were followed in this study. The rats were randomly divided into five groups: G1 served as the normal control, G2 and G3 received diets containing 5% and 10% *C. carandas* powder, while G4 and G5 were provided diets supplemented with 5% and 10% *C. carandas* extract. The diets prepared following the recipes reported in Table 1 were fed to the experimental rats for 28 days. *C. carandas* powder was administered through diet, while methanol extracts were given via oral gavage. Rats were closely monitored for morbidity and mortality and any abnormal physical and clinical changes such as eye color, eye orbit, loss of fur, any change in gait, excessive circling, and grooming. Monitoring of body weight was performed weekly, starting from initiation of the treatments. After completion of the feeding period, rats were anesthetized on the 28th day following 8 h fasting (feed withheld only) using chloroform administered via inhalation. Blood samples were collected by cardiac puncture, and the samples collected were respectively transferred to non‐heparinized and EDTA vials for serum chemistry and hematological evaluation. After a cardiac puncture, rats were sacrificed by clavicle dislocation.

**TABLE 1:** Composition of the standard and experimental rats' diets.

Feed	G1	G2	G3	G4	G5
Starch (g)	53	53	53	53	53
Casein (g)	18	18	18	18	18
Fiber (g)	5	5	5	5	5
Oil (mL)	10	10	10	10	10
CMC (g)	5	5	5	5	5
Mineral Mixture (g)	3	3	3	3	3
Vitamin Mixture (g)	1	1	1	1	1
Sugar (g)	5	5	5	5	5
*C. carandas* powder (g)	—	5	10	—	—
*C. carandas* extract (g)	—	—	—	5	10

*Note:* G1 = Normal control; G2, G3: Rats fed on 5% and 10% *C. carandas* powder, respectively; G4, G5: Rats fed on 5% and 10% *C. carandas* extract supplemented diet.

The collected blood samples were centrifuged to separate serum. A Blood Chemistry Analyzer (BTS‐350, BioSystems S.A. Barcelona, Spain) with Biosystem diagnostic kits was used to determine blood chemistry parameters, including glucose, liver enzymes, such as Alkaline phosphatase (ALP), Alkaline transaminase (ALT), Aspartate transaminase (AST), cholesterol, triglycerides, creatinine, total bilirubin, and urea (Ismail et al. 2018). Hematological indices measured were hemoglobin (Hb) level, red blood cells (RBCs) count, platelet count, mean corpuscular Hb (MCH), mean corpuscular Hb concentration (MCHC), mean corpuscular volume (MCV), hematocrit (HCT), packed cell volume (PCV), white blood cells (WBCs) count, lymphocytes, and neutrophils.

Randomly collected liver, kidney, and heart tissue samples from different experimental groups were fixed in 10% neutral buffered formalin for histological examination. The fixed tissues were washed with water, dehydrated in ascending grades of alcohol concentrations, cleared in xylene, and embedded in paraffin. The prepared tissue blocks were sectioned with a microtome to obtain 4–5 μm thick paraffin sections. The collected sections were mounted over the clean glass slides and fixed following published procedures of Loha et al. (2019) for hematoxylin and eosin (H&E) staining. The stained slides were examined microscopically for histological examination using a light microscope.

### 2.4 Data Analysis

Statistix 8.1 version was used for the data analysis. The data obtained were analyzed using one‐way analysis of variance (ANOVA). Results are expressed as the mean ± standard deviation (SD). Differences among the means were computed using the least significant differences (LSD) test; differences were considered significant at *p* ≤ 0.05.

## 3 Results

### 3.1 Effect of *C. carandas* Feeding on Physical Parameters of Albino Rats

**FIGURE 1:**
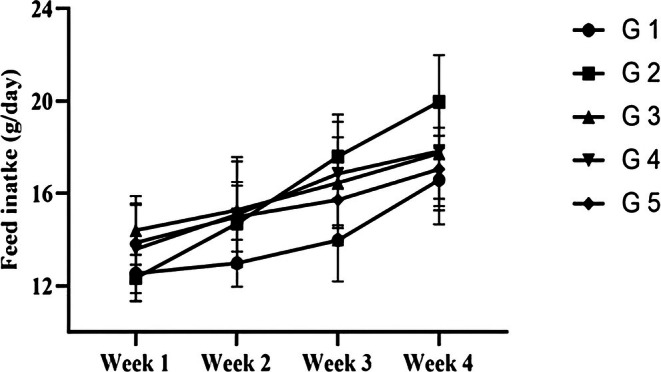
Effect of different supplemented diets on feed intake (g/day) during a 28 day feeding trial.

**FIGURE 2:**
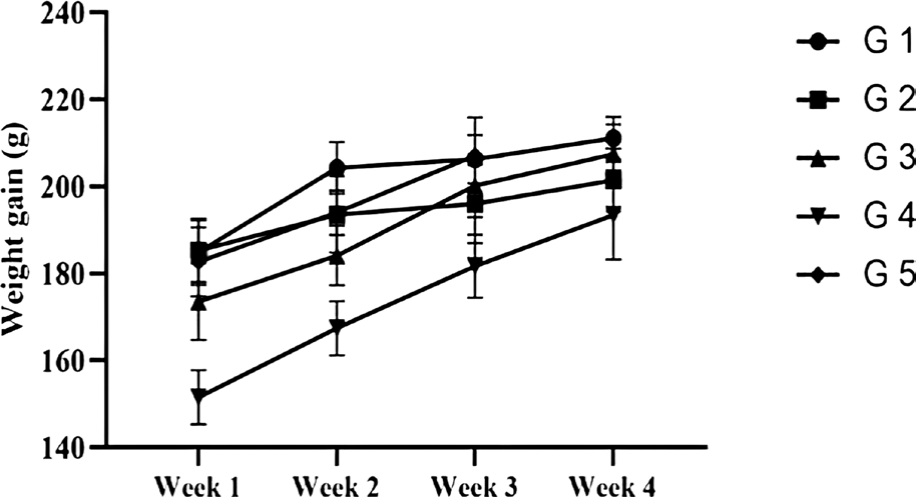
Effect of different supplemented diets on weight gain (g) for a 28‐day feeding trial. G1 = Normal control; G2, G3: Rats fed on 5% and 10% *C. carandas* powder. G4, G5: Rats fed on 5% and 10% *C. carandas* extract‐supplemented diet.

Significant variability was observed in feed intake patterns of rats fed on regular and treatment diets supplemented with 5%–10% *C. carandas* powder and extracts (Figure 1). A four‐week feeding trial showed up to 62% increase in feed intake from 37 to 59.9 g/day by the rats fed on a 5% *C. carandas* powder supplemented diet, whereas those fed on a regular diet exhibited a 32.2% increase from 37.6 to 49.7 g/day. A comparable trend was observed in the rat group fed on a 5% *C. carandas* extract‐supplemented diet. Rats in the experimental groups were given basal diets supplemented with 10% powder and extract of *C. carandas* ; these rats showed an increase of 23.2% and 23%, respectively. Sub‐acute exposure of male albino rats to *C. carandas* dried powder and extracts was studied in a regular diet at 5% and 10% supplementation levels. Potential differences in rats' growth rates were recorded with the type and concentration of the supplement (Figure 2). A significant effect of supplementation was observed on the weight of rats, suggesting 5% extracts supplementation as the most effective dietary regime to increase rat's growth up to 27.6% from the baseline, whereas a similar level of *C. carandas* powder supplementation anticipated proportionally lowest gain in weight, i.e., 8.7%.

### 3.2 Effect of *C. carandas* Supplementation on Serum Hematological Indices

Results from this investigation indicated non‐significant (*p* > 0.05) differences in nearly all hematological indices of rats fed on a regular diet and the one supplemented with varying levels of *C. carandas* powder and fruit extracts (Table 2). Moreover, the results of the hematological study were well in the range established for healthier male rats aged between 8 and 16 months. Our findings show a highly significant (*p* = 0.003) effect of sub‐acute feeding of *C. carandas* powder and extracts on the mean Hb levels of rats, with a marked increase in Hb concentration. The HB level of normal rats was 11.2 g/dL; however, nearly one to three points higher Hb levels were observed in treatment groups fed on a 5%–10% powder and extracts‐supplemented diet. The highest Hb levels, i.e., 14 and 13.3 g/dL, were recorded in 5% *C. carandas* extract, followed by powder‐supplemented diet‐fed rats, respectively. However, non‐significant (*p* > 0.05) responses were recorded on other hematological markers such as RBCs, HCT, MCH, and MCV in rats fed on varying levels of *C. carandas* extracts followed by the powder.

**TABLE 2:** Effect of *C. carandas* powder and extracts sub‐acute feeding on hematological indices of albino rat's model.

Parameter	Standard values	G1	G2	G3	G4	G5
RBC (10^6^/μL)	7.27–9.65	5.89 ± 0.90^b^	6.57 ± 0.35^ab^	6.82 ± 0.48^ab^	7.52 ± 0.63^a^	6.96 ± 0.58^ab^
Platelets (10^3^/μL)	638–1177	627 ± 205^b^	923 ± 82^ab^	788 ± 38^ab^	1090 ± 65^a^	662 ± 329^b^
Hb (g/dL)	13.7–17.6	11.2 ± 0.68^c^	13.3 ± 0.89^ab^	12.5 ± 0.12^b^	14.0 ± 0.67^a^	12.4 ± 0.49^b^
MCH (pg)	17.1–20.4	18 ± 0.40	18.6 ± 1.0	18.4 ± 1.45	18.6 ± 0.97	19.2 ± 1.1
MCHC (g/dL)	32.9–37.5	32.3 ± 0.44	32.8 ± 0.79	32.4 ± 0.44	31.9 ± 0.75	33.3 ± 2.27
MCV (fL)	48.9–57.9	55.8 ± 0.49	57.4 ± 1.66	56.6 ± 3.84	57.8 ± 1.27	57.6 ± 1.72
HCT (%)	39.6–52.5	35 ± 2.37^b^	41 ± 74^ab^	38 ± 0.15^ab^	44 ± 2.37^ab^	46 ± 11.4^a^
MPV (fL)	6.2–9.4	9.7 ± 1.21	9.3 ± 0.35	9.2 ± 0.64	9.1 ± 0.20	10.0 ± 0.95
RDW (%)	11.1–15.2	13 ± 0.85	13 ± 0.40	12 ± 1.21	12 ± 0.72	13 ± 2.61
WBC (10^3^/μL)	1.96–8.25	6.97 ± 3.60	6.67 ± 0.57	6.87 ± 0.83	3.73 ± 1.35	4.53 ± 0.91
Lymp (%)	66.6–90.3	78 ± 5.41	81 ± 0.87	80 ± 4.02	81 ± 1.37	85 ± 4.10
Neut (%)	6.2–26.7	22 ± 5.40	19 ± 0.87	20 ± 4.01	19 ± 1.36	15 ± 4.10

*Note:* Means ± SD. G1 = Normal control; G2, G3: Rats fed on 5% and 10% *C. crandas* powder; G4, G5: Rats fed on 5% and 10% *C. crandas* extract supplemented diet. Means sharing different lettering in a column differ significantly at *p* ≤ 0.05.

An association among the hematological indices was determined using the Pearson correlation. Our results suggest significant (*p* = 0.008, 0.005) and moderate positive correlation (*r* = 0.658, 0.684) between Hb and RBCs, Hb and platelet counts, respectively. Moreover, significant (*p* = 0.02) and positive correlation (*r* = 0.60, 0.58) were also recorded between HCT and RBCs, HCT and RDW, respectively. Likewise, the WBC count of the treatment groups after sub‐acute feeding was 3.73 to 6.87 × 103/μL and in the standard range established for the healthier rats, i.e., 1.96–8.25 10^3^/μL. The mean levels of the lymphocytes and the neutrophils of the rats in treatment groups were also non‐significantly different from the normal rats and were observed in standard limits established for the healthier rats.

### 3.3 Effect of *C. carandas* Supplementation on Serum Chemistry

Our findings showed non‐significant differences (*p* > 0.05) in various serological markers after feeding *C. carandas* powder and extracts‐supplemented diets to rats for 4 weeks (Table 3). In addition, results from the sub‐acute feeding study did not show any undesirable changes in the serum chemistry of the rats when compared with the control group and those of the standard values for the male albino rats' model. Serum levels of liver enzymes, including ALP, ALT, and AST, were in the range between 137 and 174, 27 and 42, and 98 and 135 U/L, respectively, and fell in standard limits, i.e., 62–230, 18–45 and 74–143 U/L for male albino Wistar rats aged 8–16 weeks. Levels of serum cholesterol and triglycerides, 55–62 and 75–96 mg/dL, respectively, were also in conformity with the standard ranges. The renal function of *C. carandas* extracts and powder‐fed rats, as evaluated by serum concentrations of creatinine (0.17–0.35 mg/dL) and urea (15–27 mg/dL) also did not differ significantly from those of the rats in the control group (Table 2). A mean serum urea concentration of 27 mg/dL was observed in the rats' group fed on 5% *C. carandas* extracts, which was non‐significantly (*p* = 0.18) higher than the standard upper limit, i.e., 24.6 mg/dL. However, serum creatinine concentration in the same group was in the normal range and at par with the values recorded for the standard control, suggesting a non‐toxicological response of sub‐acute feeding of *C. carandas* extracts and pulp powder in the renal function test. Furthermore, serum random glucose levels of the control and *C. carandas* ‐fed rats were 112–164 mg/dL, indicating that *C. carandas* did not negatively influence pancreatic function and maintained a normal glycemic response. Total bilirubin levels of the rats fed with varying levels of *C. carandas* powder and extracts were 0.12–0.13 mg/dL and at par with those recorded in the control group rats.

**TABLE 3:** Effect of *C. carandas* powder and extracts sub‐acute feeding on serum chemistry of albino rat's model.

Parameter	Std. values	G1	G2	G3	G4	G5
ALP (U/L)	62–230	169 ± 16	152 ± 20	137 ± 16	141 ± 16	174 ± 18
ALT (U/L)	18–45	42 ± 5	29 ± 6	39 ± 5	37 ± 5	27 ± 5
AST (U/L)	74–143	135 ± 13	98 ± 16	141 ± 13	101 ± 13	103 ± 13
Cholesterol (mg/dL)	37–85	55 ± 5	62 ± 6	60 ± 5	57 ± 5	58 ± 5
Creatinine (mg/dL)	0.2–0.5	0.23 ± 0.04	0.35 ± 0.05	0.20 ± 0.04	0.27 ± 0.04	0.17 ± 0.04
Glucose (mg/dL)	70–208	127 ± 17	160 ± 21	164 ± 18	157 ± 17	112 ± 17
Total bilirubin (mg/dL)	0.05–0.15	0.13 ± 0.06	0.13 ± 0.08	0.13 ± 0.07	0.12 ± 0.07	0.13 ± 0.07
Triglycerides (mg/dL)	20–114	75 ± 6	82 ± 7	78 ± 6	84 ± 6	96 ± 5
Urea	12.3–24.6	17 ± 2.8	20 ± 3.4	15 ± 2.8	27 ± 2.7	22 ± 2.9

*Note:* Means ± SE; G1 = Normal control; G2, G3: Rats fed on 5% and 10% *C. carandas* powder; G4, G5: Rats fed on 5% and 10% *C. carandas* extract supplemented diet.

### 3.4 Effect of *C. carandas* Sub‐Acute Feeding on Hepatic and Renal Tissues

**FIGURE 3:**
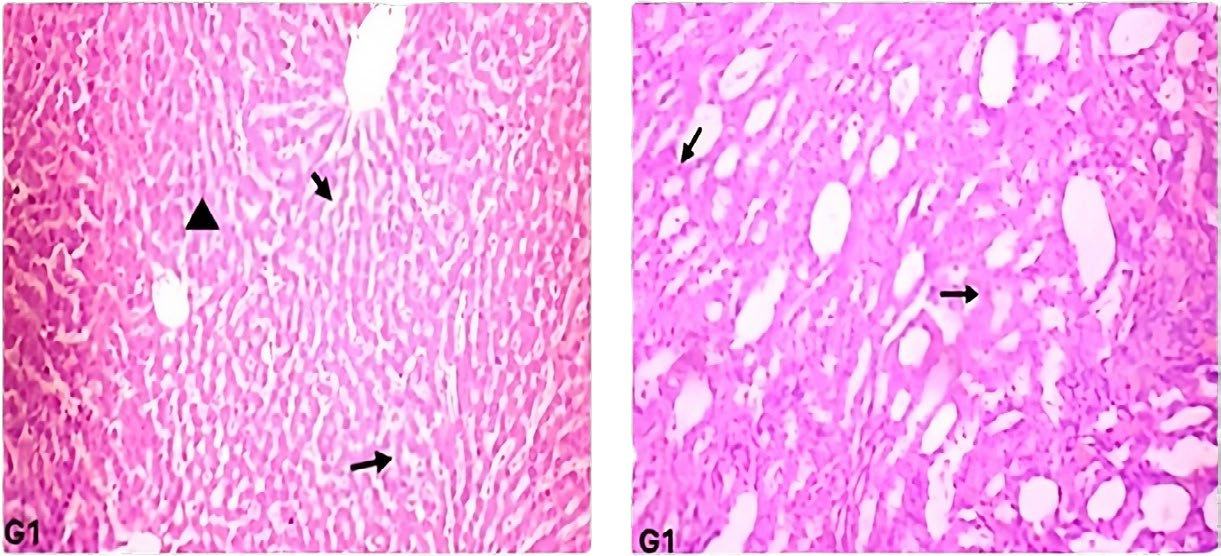
Hepatic (G1 liver) and renal histology (G1 kidney) of the rats fed on normal diet for a period of 28 days.

**FIGURE 4:**
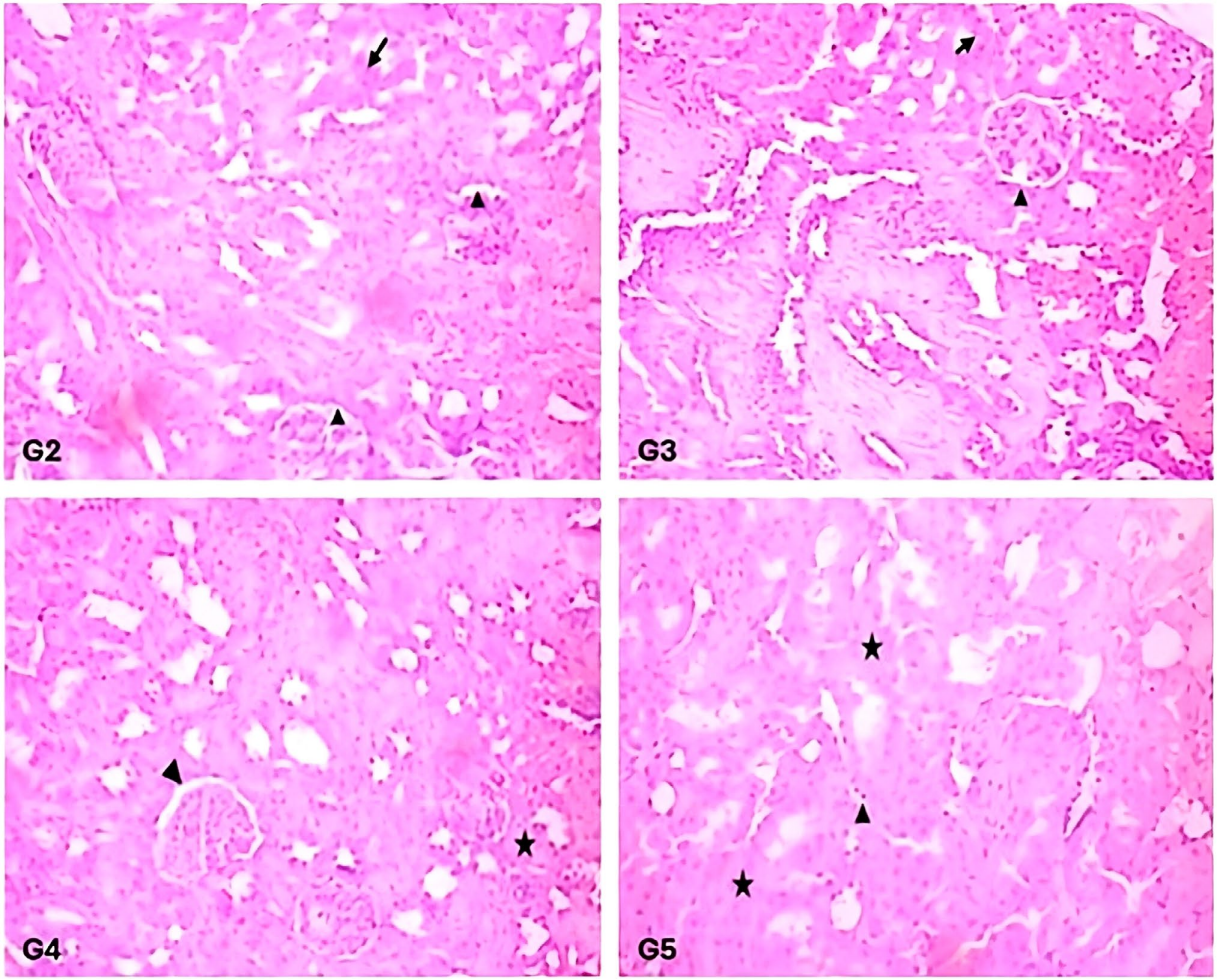
Renal histology of the rats fed with a supplemented diet after 28 days feeding trial.

**FIGURE 5:**
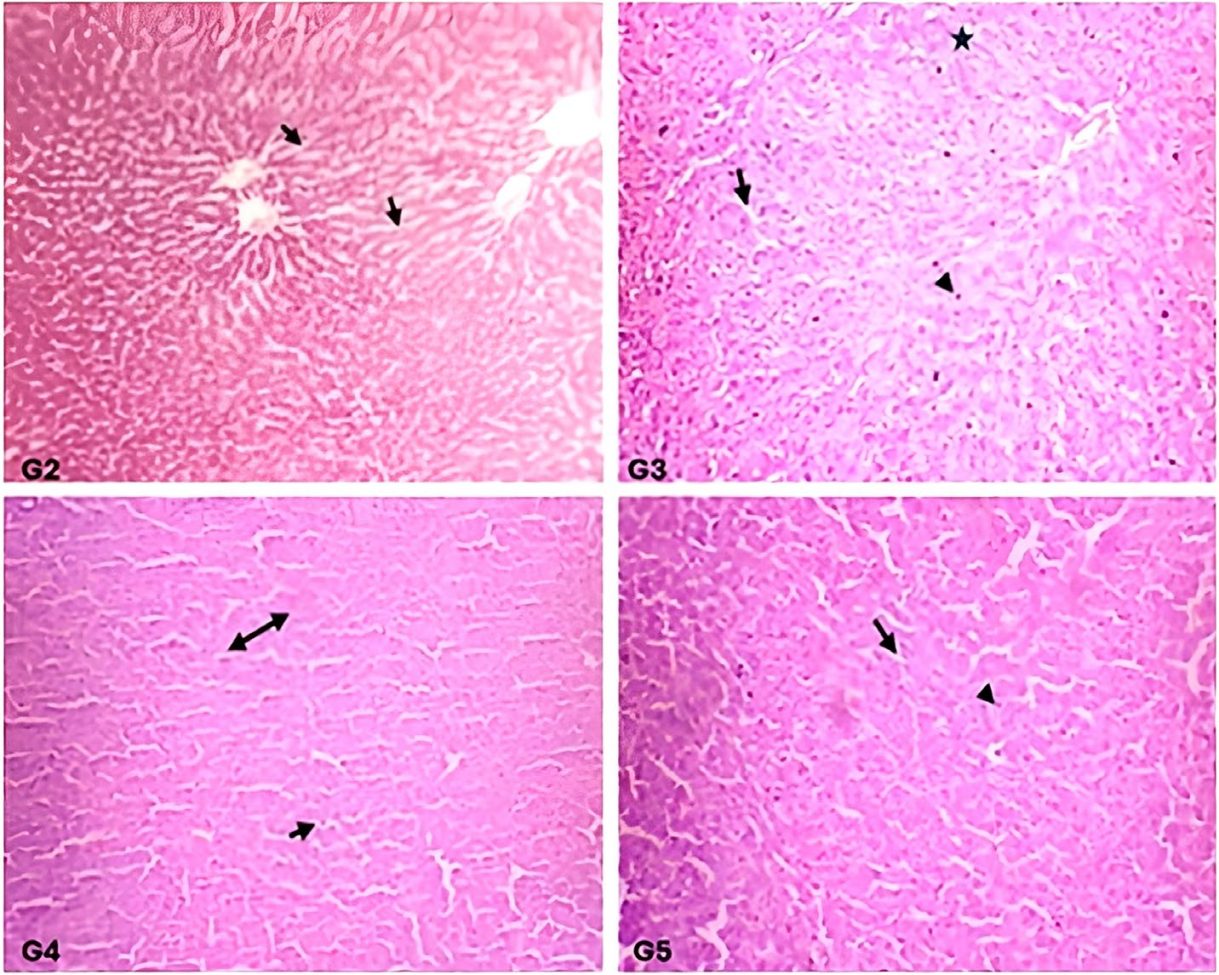
Liver histology of the rats fed with a supplemented diet after a 28 day feeding trial. G2, G3: Rats fed on 5% and 10% *C. carandas* powder; G4, G5: Rats fed on 5% and 10% *C. carandas* extract supplemented diet.

The histological examination of the liver and kidney tissues of the rats fed on a regular diet revealed intact hepatic and renal parenchyma. Results did not show any visible signs of degenerative or inflammatory response. The hepatic parenchyma of the rats in group G1 showed normal hepatocytes with centrally placed round nuclei (marked as triangle) and intact sinusoidal spaces (marked as arrow) (Figures 3, 4, 5). The histological structure of hepatic parenchyma in rats fed on a 10% *C. carandas* powder‐supplemented diet (G3) for 28 days was comparable to the control group. Despite no signs of inflammatory or degenerative changes, hepatocytes exhibited a slight necrotic change (marked as arrowhead) in a few places (Figures 3, 4, 5). Moreover, slight vacuolation (marked as steric) at certain places in the hepatic parenchyma was also observed. In the renal parenchyma, compared to the control, the cells of the tubular epithelial linings in different experimental groups offered a supplemented diet were intact with prominent nuclei with centrally placed nucleolus (marked as arrow) (Figures 3, 4, 5). The Bowman spaces of glomeruli were clear and lined by a thin rim of epithelial cells (marked as arrowhead). No prominent degenerative or inflammatory changes were observed in the renal parenchyma of experimental rats that were offered *C. carandas* powder at 5%–10% (Figures 3, 4, 5). Considering feed intake data over the 4‐week feeding study and the average weight of the rats, sub‐acute daily consumption of the *C. carandas* powder at 5%–10% supplementation levels was recorded in a range between 8.32 and 8.35 g/kg body weight (b.w).

The experimental group given *C. carandas* extracts at 5% and 10% of the diet (G4 and G5) exhibited slight changes in hepatic and renal parenchyma (Figures 3, 4, 5). The hepatic parenchyma showed a slight cellular swelling (marked as line arrow: double), as indicated by decreased sinusoidal spaces (Figures 3, 4, 5). No prominent structural change or degenerative or inflammatory response was observed in the histological study of the liver. However, the hepatic tissues of the rats fed on a 10% extracts‐supplemented diet (G5) exhibited pyknotic changes (G5) without any prominent signs of inflammatory response, except a slight congestive response observed in large blood vessels. In the renal parenchyma of the rats fed on 10%, *C. carandas* ‐supplemented extracts (G5) exhibited slight dilation of the tubular lumen and the accumulation of pink material. In some places, the glomerular spaces were reduced. However, no prominent degenerative or inflammatory response was observed except in some areas where the tubular epithelial cells exhibited a slight pyknotic response (marked as triangle). Contrary to the recorded average consumption levels of *C. carandas* powder on g/kg b.w of the rats, *C. carandas* fruit extracts consumption was 9.13 g/kg b.w. in the 5% extracts supplemented group. In contrast, it was 7.61 g/kg b.w. in the 10% extracts‐supplemented group. If correlated with the histological changes, *C. carandas* dried powder and the extracts sub‐acute consumption data indicate a higher probability of changes in the 5% extracts supplemented group followed by the tissues of *C. carandas* fruit powder‐fed rats.

## 4 Discussion

This work led to a food product development study by our group wherein 5%–10% fruit extracts and dehydrated powders of *C. carandas* were used to harness the functional and health‐promoting properties of the fruit. A sub‐acute toxicological assay was planned on the same supplementation levels to rule out the toxicological effects of the fruit and its extracts. Results of this 28‐day feeding trial reported no mortality in the experimental model at a 5%–10% level of fruit extracts and powder supplementation. Similarly, to a greater extent, the pathological investigation, as evident from the hematological and serological testing, indicates no significant changes to the vital markers, suggesting a non‐toxicological response of the experimental diets at comparatively higher doses of the fruit extracts and powders, i.e., ~9000 mg/kg b.w. However, earlier work on edible and non‐edible fractions of the neglected or underutilized fruiting plants suggests relatively lower fruit limits for acute and sub‐acute prescription as a therapeutic ingredient. Research findings Abid and Mahmood (2019) reported LD50 of *Cassia fistula* extracts as above 5000 mg/kg b.w. In contrast, ~1000 mg/kg sub‐acute exposure of the *C. fistula* extracts did not significantly affect the treatment groups' relative weight, hematological, and biochemical indices when compared with the control. In another study wherein cashew extracts were provided to the rats at 2000 mg/kg b.w, the biochemical analysis of renal and hepato‐biliary functions as indicated by serum creatinine and liver enzyme levels were found well in normal ranges, suggesting the given doses as tolerable in 30–days sub‐acute toxicity assessment model (Konan et al. 2007).

In addition, our results report a significant increase in Hb levels of the rats compared with those of control at 5%–10% extracts and powder supplementation. The Hb levels in the 5% extract group are positively correlated with RBC count (*r* = 0.658, *p* = 0.008), suggesting that the high iron content of *C. carandas* (~61 mg/100 g) effectively supports erythropoiesis. Crucially, the significant increase in platelet count (from 627 to 1090 × 10^3^ μL) and its moderate positive correlation with Hb (*r* = 0.684, *p* = 0.005) indicates that *C. carandas* may stimulate thrombocytopoiesis alongside general hematopoiesis. While earlier studies on other fruits like apple have been cited for addressing thrombocytopenia (Abbas et al. 2018), our findings provide the first sub‐acute evidence for *C. carandas* fruit powder in a dietary model. This suggests that the fruit can be exploited as a functional food candidate for disorders involving platelet deficiency without eliciting toxicological risks.

Food materials or ingredients of food origins intended for functional or therapeutic uses require deeper evaluation for their safety using standard toxicological methods. The serum chemistry results of *C. carandas‐*fed rat groups were statistically non‐significantly (*p* ≤ 0.05) different from the control group rats. However, certain parameters like ALP, ALT, and AST witnessed depression in concentration compared with the control group. Similarly, a few parameters, such as serum creatinine, urea, and glucose concentrations, were also found at peak normal values (*p* > 0.05) in groups that received the highest amount of extracts in 28‐day feeding trials. Non‐significantly lower levels of liver enzymes and the bilirubin concentration in *C. carandas* extracts and powder‐fed rats compared with the control do not establish any correlation to slight histological changes observed in the hepatic tissues of the rats in the same study groups. Based on physical, hematological, and serological findings, the sub‐chronic 5000 mg/kg/day feeding dose of the red dragon fruit methanolic extracts was considered safer in the rats' models. The results suggest non‐significant changes in body and organ weights and in liver function markers such as ALT, ALP, AST, and total bilirubin of the experimental groups when compared with the control (Hor et al. 2012). The fruit of *C. carandas* has been extensively explored for its therapeutic properties against renal and hepatic disorders. Our results suggest the protective effect of *C. carandas* fruit and its extracts against nephropathy and hepatic toxicity. It is predominantly linked with the fruit's antioxidant properties and effective phenolic and flavonoid contents (Singh and Agarwal 2022; Dhodi et al. 2015). The given protective properties of the fruit of *C. carandas* also provide the basis for its protective role against renal and hepatic disorders.

Less‐known fruits of regional significance have been explored for their possible toxicological effects on consumers. Published research of Aba and Amadi (2020) indicated mild renal and hepatic toxicology effects of *Averrhoa carambola* fruit juice without visible signs of toxicity on hematological indices. The referred study reports lesions of degeneration and necrosis as histomorphological changes in the liver and kidney tissues of rats fed on *A. carambola* fruit juice (~5000 mg/kg b.w.) for 28 days. In another study of Nuru et al. (2024), researchers reported 2000 mg/kg b.w. feeding *Ziziphus spina‐Christi* fruit extracts to rats results in the dissolution of nuclear and cytoplasmic components, resulting in generalized tissue degeneration and glomerular atrophy. Our study, however, did not show degeneration or necrosis of hepatic and renal tissues at relatively higher feeding levels. Likewise, serological markers such as liver enzymes, such as ALT, AST, ALP, concentration of bilirubin, and serum creatinine were found well in the normal ranges and non‐significantly different from the indices recorded for the control rats. This indicates a weaker correlation between the serological index and the changes recorded in hepatic and renal tissues of the rats provided with *C. carandas* extracts and powder‐supplemented diets.

*C. carandas* fruit has been reported as a promising carrier of phytochemicals of health significance, such as anthocyanins, phenolic acids, flavonoids, and fatty acid esters, among others, that may exhibit a wide range of biological activities (Anupama et al. 2014; Sarkar et al. 2018; Kiruthika et al. 2019). A study citing the anti‐inflammatory properties of *C. carandas* extract reported 76% inhibition of carrageenan‐induced inflammation on an exposure of 400 mg/kg b.w. fruit extracts (Anupama et al. 2014). Another rat study using *C. carandas* fruit extracts at the rate of 300 mg/kg b.w. reported antidiabetic properties of the fruit extracts with 46%–70% reduction in blood glucose and glycosylated Hb levels (Singh et al. 2019). Furthermore, the anticancer effects of *C. carandas* fruit extracts have also been documented against cancer cell lines of cervical, breast, hepatocellular, and bone cancers with IC50 in a range between 56.72 and 82.90 μg/mL (Gupta et al. 2014).

Looking into the prospective health benefits associated with the consumption of *C. carandas* fruit and its extracts as cited above, acute to sub‐acute feeding of the fruit at a level below 9000 mg/kg b.w. as has been practiced in a recent study may serve as a therapeutic role without anticipating significant toxicological effects.

## 5 Limitations

The present sub‐acute (28 day) feeding study of 5%–10% *C. carandas* dehydrated powder and methanolic extracts to male rats found no evidence of toxicity based on physical observations, hematology and serology, but only slight, non‐significant and weakly correlated histological changes in liver and kidney tissues and no inflammatory or degenerative lesions. However, the study did not investigate mechanisms of toxicity or toxicokinetics, such as iron metabolism biomarkers, erythropoietin or thrombopoietin pathways, metabolite profiling, or ADME, and was further limited to a short exposure period and a single sex. Therefore, future studies should utilize longer‐term exposure, include both sexes, analytically confirm the composition of the extracts and residual solvent levels, and incorporate targeted mechanistic endpoints to elucidate the observed hematological effects.

## Author Contributions

**Raheel Suleman:** writing – review and editing. **Rai Muhammad Amir:** writing – review and editing. **Sheraz Ahmed Bhatti:** writing – review and editing. **Robert Mugabi:** writing – review and editing, software. **Muhammad Waseem:** formal analysis, writing – review and editing. **Wisha Saeed:** formal analysis, acquisition of data, writing first draft. **Muhammad Qamar:** conceptualization, supervision. **Asher Abdur Rehman:** acquisition of data. **Iqra Akram:** formal analysis. **Muhammad Zulqarnain Khan:** graphics, materials and methodology. **Tariq Ismail:** conceptualization, supervision and resources, correspondence.

## Funding

The authors have nothing to report.

## Ethics Statement

The research protocols of this study were approved by the Bioethical Committee (Approval # 02–23) of the Faculty of Food Science and Nutrition, Bahauddin Zakariya University Multan, Pakistan.

## Conflicts of Interest

The authors declare no conflicts of interest.

## Data Availability

The data that support the findings of this study are available from the corresponding author upon reasonable request.
